# Crystal and mol­ecular structure of 4-fluoro-1*H*-pyrazole at 150 K

**DOI:** 10.1107/S2056989023003055

**Published:** 2023-04-06

**Authors:** Basil M. Ahmed, Matthias Zeller, Gellert Mezei

**Affiliations:** a Western Michigan University, Department of Chemistry, 1903 W. Michigan Ave., Kalamazoo, MI 49008, USA; b Purdue University, Department of Chemistry, 101 Wetherill Hall (WTHR), 560 Oval Drive, West Lafayette, IN 47907, USA; Katholieke Universiteit Leuven, Belgium

**Keywords:** pyrazole, crystal structure, low temperature, hydrogen-bonding motifs

## Abstract

Only two 4-halo-1*H*-pyrazole crystal structures are known to date (chloro and bromo, the structure of 4-iodo-1*H*-pyrazole has not been reported yet). The triclinic structure of 4-fluoro-1*H*-pyrazole, C_3_H_3_FN_2_ (*P*  ), reported here is not isomorphous with those of the chloro and bromo analogues (which are isomorphous, ortho­rhom­bic *Pnma*). To avoid sublimation during the measurement, diffraction data were collected at 150 K. Two crystallographically unique 4-fluoro-1*H*-pyrazole moieties linked by an N—H⋯N hydrogen bond are found in the asymmetric unit. Unlike the trimeric supra­molecular motifs found in the structures of the chloro and bromo analogues, 4-fluoro-1*H*-pyrazole forms one-dimensional chains by inter­molecular hydrogen bonding in the crystal.

## Abstract 2

Two crystallographically unique 4-fluoro-1*H*-pyrazole moieties linked by an N—H⋯N hydrogen bond are found in the asymmetric unit. Unlike the trimeric supra­molecular motifs found in the structures of the chloro and bromo analogues, 4-fluoro-1*H*-pyrazole forms one-dimensional chains by inter­molecular hydrogen bonding in the crystal.

## 1. Chemical context

**Figure 1:**
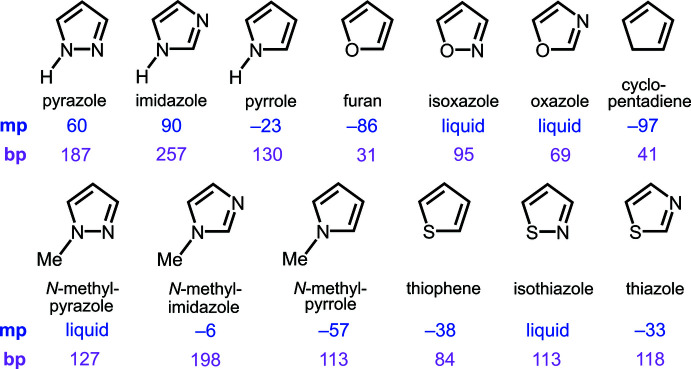
Comparison of the structures of five-membered aromatic heterocycles and their corresponding melting and boiling points (°C) according to the *CRC Handbook of Chemistry and Physics* (Rumble, 2022 ▸) or the literature. Those with no melting points reported are liquids at room temperature.

1*H*-Pyrazole (pzH) is both a hydrogen-bond donor and acceptor mol­ecule, owing to its NH and N centers. Consequently, pyrazole moieties of the parent compound or C-substituted analogues form hydrogen bonds to each other in the corresponding crystal structures, and similarly to imidazole, have higher melting and boiling points than other five-membered cyclic aromatic mol­ecules lacking either the hydrogen-bond acceptor (pyrrole), the hydrogen-bond donor (*N*-methyl derivatives, furan, isoxazole, oxazole, thio­phene, iso­thia­zole, thia­zole) or both centers (cyclo­penta­diene) (Fig. 1 ▸). The proximity of the hydrogen-bond donor and acceptor centers in pz allows for the formation of either discreet hydrogen-bonded motifs, such as dimers, trimers, tetra­mers and hexa­mers, or polymeric catemers depending on the substituents (Bertolasi *et al.*, 1999 ▸; Foces-Foces *et al.*, 2000 ▸; Claramunt *et al.*, 2006 ▸; Alkorta *et al.*, 2006 ▸), whereas imidazole only forms catemers (Cammers & Parkin, 2004 ▸). 
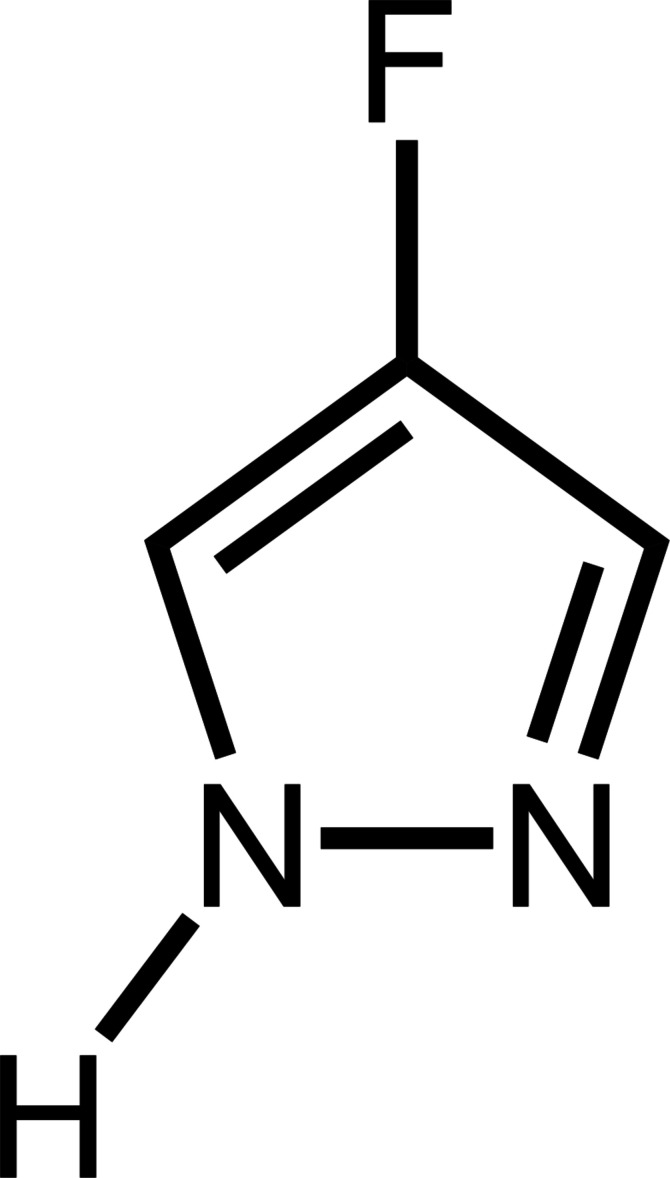


## 2. Structural commentary

**Figure 2:**
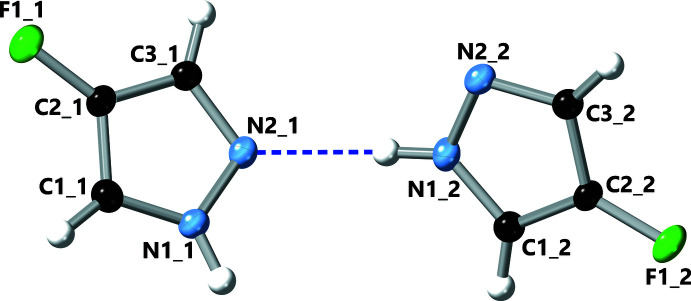
Displacement ellipsoid plot (50% probability) of the crystal structure of 4-fluoro-1*H*-pyrazole, showing the contents of the asymmetric unit.

As shown in Fig. 2 ▸, the asymmetric unit contains two symmetry-independent 4-fluoro-1*H*-pyrazole moieties (*Z*′ = 2; *P* 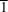 ) , similarly to 1*H*-pyrazole (*Z*′ = 2; *P*2_1_ *cn*/*Pna*2_1_; La Cour & Rasmussen, 1973 ▸; Sikora & Katrusiak, 2013 ▸) but unlike the chloro and bromo analogues (*Z*′ = 1.5; *Pnma*) (Rue & Raptis, 2021 ▸; Foces-Foces *et al.*, 1999 ▸). Structures with *Z*′ >1 result when two or more inter­molecular inter­actions, such as optimal shape packing, optimization of hydrogen bonds and aromatic inter­actions, are in conflict (Steed & Steed, 2015 ▸). Therefore, the *Z*′ value larger than 1 observed in these pyrazole structures emphasizes the importance of hydrogen bonding in their solid-state structures.

The two crystallographically independent 4-Fpz moieties, which are identical within experimental error, are planar with deviations from the C_3_FN_2_ mean-plane of less than 0.004 and 0.008 Å, respectively. Table 1 ▸ presents a comparison of bond lengths determined by X-ray diffraction for the parent pzH at 150 K (Sikora & Katrusiak, 2013 ▸), 4-FpzH at 150 K, 4-ClpzH at 150 K (Rue & Raptis, 2021 ▸) and 4-BrpzH at room temperature (Foces-Foces *et al.*, 1999 ▸). All structures contain two symmetry-independent moieties. In the case of pzH and 4-FpzH, the NH and N centers of the pz rings are distinct, unlike in the case of 4-Cl/BrpzH. Therefore, in the former case two distinct sets of C—N and C—C bond distances are observed. Similarly to pzH, in 4-FpzH the C—N bond adjacent to N is shorter than the one adjacent to NH [by 0.008 (1)/0.010 (1) Å], whereas the C—C bond adjacent to N is longer than the one adjacent to NH [by 0.019 (1)/0.018 (1) Å]. In general, the N—N, C—N and C—C bond distances in 4-*R*pH are consistent across the *R* = H, F, Cl and Br series.

## 3. Supra­molecular features

**Figure 3:**
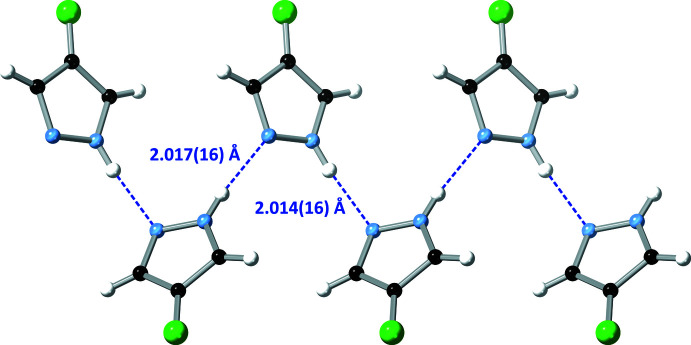
View (along the *b* axis) of one of the hydrogen-bonded chains in the crystal of 4-fluoro-1*H*-pyrazole.

**Figure 4:**
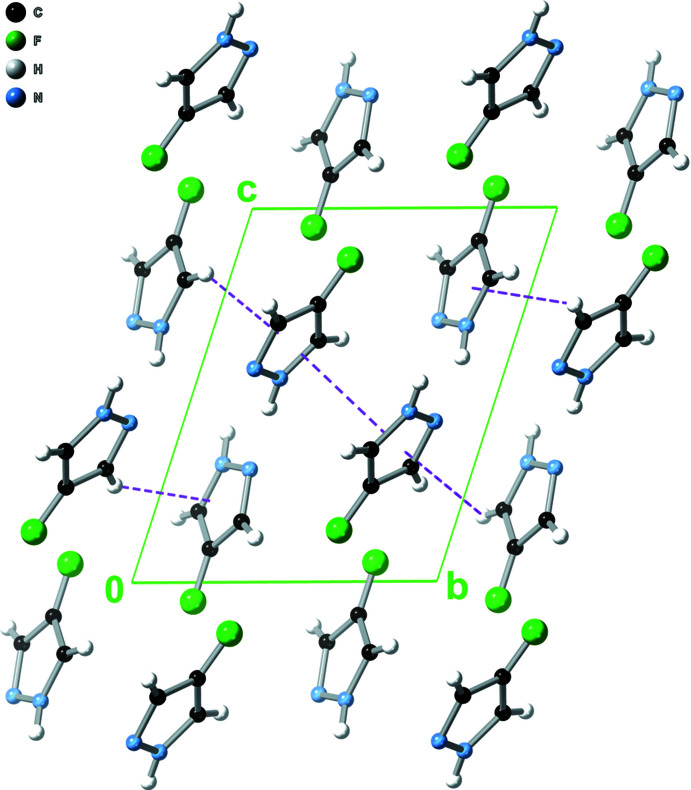
Packing diagram (along the *a* axis) of 4-fluoro-1*H*-pyrazole, showing both π–π and C—H⋯π inter­actions.

**Figure 5:**
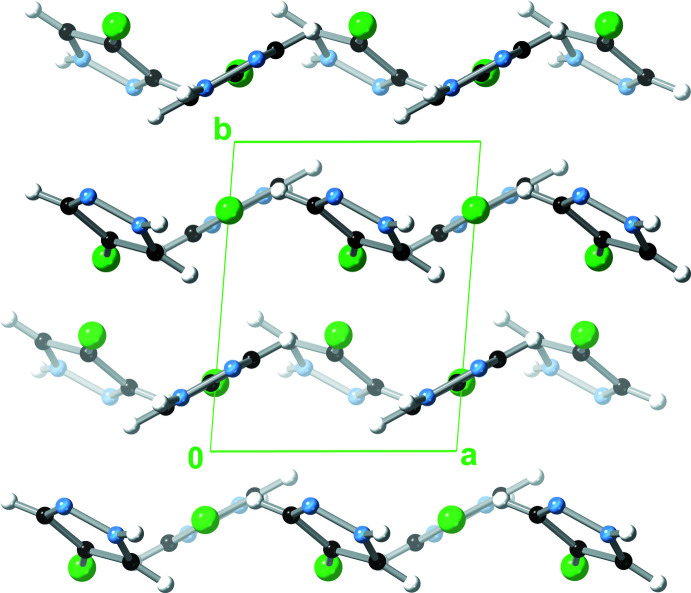
Packing diagram (along the *c* axis) of 4-fluoro-1*H*-pyrazole, showing the herringbone-type packing of the pyrazole moieties.

The pz N—H proton donates an N—H⋯N hydrogen bond to a neighbouring pz unit on one side, while the pz N atom accepts an N—H⋯N hydrogen bond from another pz unit on the opposite side (Table 2 ▸, Fig. 3 ▸). Thus, pz units in the resulting 4-FpzH catemer form dihedral angles of 59.74 (3)° with each other, with centroid–centroid distances of 4.9487 (5) Å. Adjacent catemers inter­act with each other by π–π stacking [distance between pz mean-planes = 3.4911 (8) Å; dihedral angle between pz mean planes = 0°, crystallographically imposed; centroid–centroid distance = 3.7034 (6) Å] and C—H⋯π inter­actions [dihedral angle between pz mean planes = 59.74 (3)°; centroid–centroid distance = 4.3794 (5)/4.4791 (5) Å, H⋯centroid distance = 2.8030 (3)/2.9007 (4) Å], on alternate sides (Fig. 4 ▸). As a result, the pz units are arranged in a herringbone pattern within the crystal (Fig. 5 ▸).

## 4. Database survey

**Figure 6:**
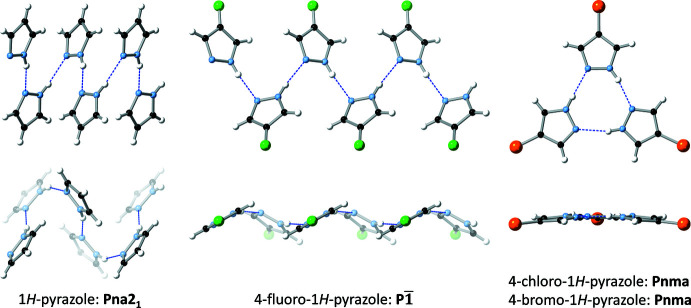
Comparison of the hydrogen-bonded motifs (two different views for each) in the crystal packing of 4-*R*pzH (*R* = H, F, Cl and Br).

Although the parent pyrazole (pzH) also forms a hydrogen-bonded catemer in the crystal packing, its structure is quite different from the one formed by 4-FpzH. As illustrated in Fig. 6 ▸, pairs of pz units are found in two different geometries in the catemer of pzH: one in which they form dihedral angles of 5.45 (9)° with centroid–centroid distances of 5.1659 (15) Å, and another with dihedral angles of 74.64 (9)° and centroid–centroid distances of 5.0504 (15) Å. In contrast, pz units in 4-Cl/BrpzH form discreet hydrogen-bonded trimers. While the presence/nature of the 4-halo substituent leads to very different outcomes in terms of the overall packing and hydrogen-bonded motifs in 4-*R*pzH (*R* = H, F, Cl/Br), it has little effect on the corresponding hydrogen bonding parameters (Table 2 ▸). Inter­estingly, the structure of the catemer in 4-FpzH is essentially identical to the one found in the lattice of 4-acetyl-1*H*-pyrazole (monoclinic *P*2_1_/*n*; Frey *et al.*, 2014 ▸), with dihedral angles of 57.28 (7)° and centroid–centroid distances of 4.9501 (13) Å between adjacent pz units. The structure of the different catemer of pzH is also found in the crystal lattices of 4-phenyl-1*H*-pyrazole (ortho­rhom­bic, *Pbcn*; Reger *et al.*, 2003 ▸) and 4-adamantyl-1*H*-pyrazole (triclinic, *P* 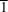 ; Cabildo *et al.*, 1994 ▸), despite the large substituents. 4-Methyl-1*H*-pyrazole (ortho­rhom­bic, *Pca*2_1_; Goddard *et al.*, 1999 ▸) and 4-nitro-1*H*-pyrazole (triclinic, *P* 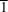 ; Llamas-Saiz *et al.*, 1994 ▸), on the other hand, form trimers, similarly to 4-Cl/BrpzH.

## 5. Synthesis and crystallization

4-Fluoro-1*H*-pyrazole was synthesized according to a published procedure, from sodium fluoro­acetate (*WARNING: highly toxic!*) by reaction with oxalyl chloride and di­methyl­formamide, followed by treatment with base and then hydrazine (England *et al.*, 2010 ▸). Single crystals were obtained from the powder by slow isothermal sublimation inside a sealed vial under ambient conditions.

## 6. Refinement

Crystal data, data collection and structure refinement details are summarized in Table 3 ▸. C—H bond distances were constrained to 0.95 Å and these H atoms were refined as riding. Positions of N-bound H atoms were freely refined. *U*_iso_(H) values were set to 1.2 times *U*_eq_(C/N) for all H atoms.

## Supplementary Material

Crystal structure: contains datablock(s) I. DOI: 10.1107/S2056989023003055/vm2280sup1.cif

Structure factors: contains datablock(s) I. DOI: 10.1107/S2056989023003055/vm2280Isup2.hkl

CCDC reference: 2253642

Additional supporting information:  crystallographic information; 3D view; checkCIF report

## Figures and Tables

**Table 1:** Comparison of N—N, C—N and C—C bond lengths determined by X-ray diffraction in 4-*R*pzH (*R* = H, F, Cl and Br) Numbers marked with an asterisk indicate average values in the case of pyrazoles with disordered NH/N centers.

	N—N	C—N (NH)	C—N (N)	C—C (NH)	C—C (N)
pzH^*a*^	1.354 (2)	1.337 (3)	1.330 (3)	1.368 (3)	1.389 (3)
	1.351 (2)	1.344 (3)	1.326 (3)	1.366 (3)	1.389 (3)
4-FpzH^*b*^	1.3484 (9)	1.3473 (10)	1.3391 (10)	1.3729 (11)	1.3922 (11)
	1.3513 (10)	1.3476 (10)	1.3375 (10)	1.3742 (10)	1.3924 (10)
4-ClpzH^*c*^	1.346 (2)	1.335 (2)*	1.334 (2)*	1.380 (2)*	1.374 (2)*
	1.345 (3)	1.334 (2)*	1.334 (2)*	1.377 (2)*	1.377 (2)*
4-BrpzH^*d*^	1.335 (9)	1.327 (10)*	1.331 (10)*	1.391 (11)*	1.338 (10)*
	1.335 (9)	1.343 (10)*	1.343 (10)*	1.371 (9)*	1.371 (9)*

Notes: (*a*) Sikora & Katrusiak (2013 ▸); (*b*) this work; (*c*) Rue & Raptis (2021 ▸); (*d*) Foces-Foces *et al.* (1999 ▸).

**Table 2:** Hydrogen-bond geometry (Å, °) for 4-*R*pzH (*R* = H, F, Cl and Br)

*D*—H⋯*A*	*D*—H	H⋯*A*	*D*⋯*A*	*D*—H⋯*A*
pzH				
N1—H1⋯N3	0.860 (2)	2.038 (2)	2.885 (3)	167.73 (10)
N4—H5⋯N2^i^	0.861 (2)	2.083 (2)	2.881 (3)	153.87 (13)
4-FpzH				
N1_2—H1*N*_2⋯N2_1	0.878 (14)	2.014 (16)	2.8764 (10)	166.6 (13)
N1_1—H1*N*_1⋯N2_2^ii^	0.892 (14)	2.017 (16)	2.9024 (10)	172.4 (15)
4-ClpzH				
N1—H1*A*⋯N1^iii^	0.88	2.03	2.885 (3)	165
N2—H2⋯N3^iv^	0.88	1.99	2.8582 (19)	169
N3—H3*A*⋯N2^iv^	0.88	1.99	2.8582 (19)	169
4-BrpzH				
N12—H12⋯N21	1.02	1.87	2.871 (9)	169
N21—H21⋯N12	1.01	1.87	2.871 (9)	171
N22—H22⋯N22^v^	1.02	1.93	2.922 (9)	164

Symmetry codes: (i) *x* + 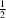 , −*y* + 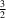 , *z*; (ii) *x* + 1, *y*, *z*; (iii) *x*, −*y* + 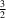 , *z*; (iv) −*x* + 1, −*y* + 1, −*z*; (v) *x*, −*y* + 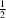 , *z*.

**Table 3:** Experimental details

Crystal data	
Chemical formula	C_3_H_3_FN_2_
*M*_r_	86.07
Crystal system, space group	Triclinic, *P* 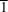
Temperature (K)	150
*a*, *b*, *c* (Å)	5.6045 (2), 7.4315 (3), 9.5396 (4)
α, β, γ (°)	71.689 (2), 87.731 (2), 84.968 (2)
*V* (Å^3^)	375.72 (3)
*Z*	4
Radiation type	Mo *K*α
μ (mm^−1^)	0.14
Crystal size (mm)	0.45 × 0.43 × 0.38

Data collection	
Diffractometer	Bruker AXS D8 Quest
Absorption correction	Multi-scan (*SADABS*; Krause *et al.*, 2015 ▸)
*T*_min_, *T*_max_	0.678, 0.747
No. of measured, independent and observed [*I* > 2σ(*I*)] reflections	15049, 2875, 2635
*R*_int_	0.024
(sin θ/λ)_max_ (Å^−1^)	0.770

Refinement	
*R*[*F* ^2^ > 2σ(*F* ^2^)], *wR*(*F* ^2^), *S*	0.036, 0.108, 1.06
No. of reflections	2875
No. of parameters	116
H-atom treatment	H atoms treated by a mixture of independent and constrained refinement
Δρ_max_, Δρ_min_ (e Å^−3^)	0.47, −0.26

Computer programs: *APEX4* and *SAINT* (Bruker, 2022 ▸), *SHELXT* (Sheldrick, 2015*a* ▸), *SHELXL2018/3* (Sheldrick, 2015*b* ▸) and *ShelXle* Rev1460 (Hübschle *et al.*, 2011 ▸).

## supplementary crystallographic information

### Crystal data

**Table:**

C_3_H_3_FN_2_	*Z* = 4
*M**_r_* = 86.07	*F*(000) = 176
Triclinic, *P*1	*D*_x_ = 1.522 Mg m^−^^3^
*a* = 5.6045 (2) Å	Mo *K*α radiation, λ = 0.71073 Å
*b* = 7.4315 (3) Å	Cell parameters from 9943 reflections
*c* = 9.5396 (4) Å	θ = 2.3–33.1°
α = 71.689 (2)°	µ = 0.14 mm^−^^1^
β = 87.731 (2)°	*T* = 150 K
γ = 84.968 (2)°	Block, colourless
*V* = 375.72 (3) Å^3^	0.45 × 0.43 × 0.38 mm

### Data collection

**Table:**

Bruker AXS D8 Quest diffractometer	2875 independent reflections
Radiation source: fine focus sealed tube X-ray source	2635 reflections with *I* > 2σ(*I*)
Triumph curved graphite crystal monochromator	*R*_int_ = 0.024
Detector resolution: 7.4074 pixels mm^-1^	θ_max_ = 33.2°, θ_min_ = 2.3°
ω and phi scans	*h* = −8→8
Absorption correction: multi-scan (SADABS; Krause *et al.*, 2015)	*k* = −11→11
*T*_min_ = 0.678, *T*_max_ = 0.747	*l* = −14→14
15049 measured reflections	

### Refinement

**Table:**

Refinement on *F*^2^	Secondary atom site location: difference Fourier map
Least-squares matrix: full	Hydrogen site location: mixed
*R*[*F*^2^ > 2σ(*F*^2^)] = 0.036	H atoms treated by a mixture of independent and constrained refinement
*wR*(*F*^2^) = 0.108	*w* = 1/[σ^2^(*F*_o_^2^) + (0.0579*P*)^2^ + 0.0788*P*] where *P* = (*F*_o_^2^ + 2*F*_c_^2^)/3
*S* = 1.06	(Δ/σ)_max_ < 0.001
2875 reflections	Δρ_max_ = 0.47 e Å^−^^3^
116 parameters	Δρ_min_ = −0.26 e Å^−^^3^
0 restraints	Extinction correction: *SHELXL2018/3* (Sheldrick 2015b), Fc^*^=kFc[1+0.001xFc^2^λ^3^/sin(2θ)]^-1/4^
Primary atom site location: dual	Extinction coefficient: 0.040 (10)

### Special details

**Table:**

Geometry. All esds (except the esd in the dihedral angle between two l.s. planes) are estimated using the full covariance matrix. The cell esds are taken into account individually in the estimation of esds in distances, angles and torsion angles; correlations between esds in cell parameters are only used when they are defined by crystal symmetry. An approximate (isotropic) treatment of cell esds is used for estimating esds involving l.s. planes.

### Fractional atomic coordinates and isotropic or equivalent isotropic displacement parameters (Å^2^)

**Table:**

	*x*	*y*	*z*	*U*_iso_*/*U*_eq_	
F1_1	0.51610 (12)	0.62514 (11)	0.13759 (7)	0.03931 (17)	
N1_1	0.64380 (12)	0.73038 (10)	0.45438 (7)	0.02162 (13)	
H1N_1	0.721 (3)	0.7343 (19)	0.5331 (15)	0.032*	
N2_1	0.42422 (12)	0.82300 (10)	0.42828 (7)	0.02262 (14)	
C1_1	0.71303 (14)	0.64372 (11)	0.35314 (9)	0.02302 (15)	
H1_1	0.859931	0.571209	0.349074	0.028*	
C2_1	0.52537 (14)	0.68285 (11)	0.25714 (8)	0.02251 (15)	
C3_1	0.34891 (13)	0.79346 (11)	0.30642 (8)	0.02192 (15)	
H3_1	0.198692	0.840571	0.260776	0.026*	
F1_2	0.00161 (11)	0.77865 (9)	1.05091 (6)	0.03386 (15)	
N1_2	0.13614 (12)	0.82593 (10)	0.68400 (7)	0.02239 (14)	
H1N_2	0.217 (3)	0.845 (2)	0.6004 (16)	0.034*	
N2_2	−0.06438 (12)	0.73275 (10)	0.69835 (7)	0.02286 (14)	
C1_2	0.19488 (14)	0.85549 (11)	0.81020 (8)	0.02229 (15)	
H1_2	0.328762	0.916175	0.826384	0.027*	
C2_2	0.01926 (14)	0.77888 (11)	0.91040 (8)	0.02065 (14)	
C3_2	−0.13824 (14)	0.70401 (11)	0.83817 (8)	0.02168 (14)	
H3_2	−0.276718	0.642113	0.881490	0.026*	

### Atomic displacement parameters (Å^2^)

**Table:**

	*U*^11^	*U*^22^	*U*^33^	*U*^12^	*U*^13^	*U*^23^
F1_1	0.0372 (3)	0.0597 (4)	0.0370 (3)	−0.0145 (3)	0.0046 (2)	−0.0357 (3)
N1_1	0.0213 (3)	0.0267 (3)	0.0166 (3)	−0.0024 (2)	−0.0017 (2)	−0.0061 (2)
N2_1	0.0220 (3)	0.0284 (3)	0.0182 (3)	−0.0005 (2)	0.0008 (2)	−0.0090 (2)
C1_1	0.0207 (3)	0.0237 (3)	0.0261 (3)	−0.0022 (2)	0.0014 (2)	−0.0098 (3)
C2_1	0.0232 (3)	0.0278 (3)	0.0213 (3)	−0.0083 (3)	0.0027 (2)	−0.0131 (3)
C3_1	0.0197 (3)	0.0276 (3)	0.0190 (3)	−0.0036 (2)	−0.0007 (2)	−0.0075 (2)
F1_2	0.0395 (3)	0.0492 (3)	0.0174 (2)	−0.0117 (2)	0.00202 (19)	−0.0149 (2)
N1_2	0.0231 (3)	0.0266 (3)	0.0183 (3)	−0.0023 (2)	0.0033 (2)	−0.0085 (2)
N2_2	0.0236 (3)	0.0275 (3)	0.0196 (3)	−0.0021 (2)	−0.0016 (2)	−0.0102 (2)
C1_2	0.0210 (3)	0.0261 (3)	0.0216 (3)	−0.0044 (2)	0.0006 (2)	−0.0094 (3)
C2_2	0.0228 (3)	0.0248 (3)	0.0156 (3)	−0.0029 (2)	−0.0004 (2)	−0.0078 (2)
C3_2	0.0208 (3)	0.0256 (3)	0.0200 (3)	−0.0044 (2)	0.0002 (2)	−0.0082 (2)

### Geometric parameters (Å, º)

**Table:**

F1_1—C2_1	1.3428 (8)	F1_2—C2_2	1.3396 (8)
N1_1—C1_1	1.3473 (10)	N1_2—C1_2	1.3476 (10)
N1_1—N2_1	1.3484 (9)	N1_2—N2_2	1.3513 (10)
N1_1—H1N_1	0.892 (14)	N1_2—H1N_2	0.878 (14)
N2_1—C3_1	1.3391 (10)	N2_2—C3_2	1.3375 (10)
C1_1—C2_1	1.3729 (11)	C1_2—C2_2	1.3742 (10)
C1_1—H1_1	0.9500	C1_2—H1_2	0.9500
C2_1—C3_1	1.3922 (11)	C2_2—C3_2	1.3924 (10)
C3_1—H3_1	0.9500	C3_2—H3_2	0.9500
			
C1_1—N1_1—N2_1	112.75 (6)	C1_2—N1_2—N2_2	112.72 (6)
C1_1—N1_1—H1N_1	129.5 (9)	C1_2—N1_2—H1N_2	129.9 (9)
N2_1—N1_1—H1N_1	117.7 (9)	N2_2—N1_2—H1N_2	116.9 (9)
C3_1—N2_1—N1_1	105.50 (6)	C3_2—N2_2—N1_2	105.43 (6)
N1_1—C1_1—C2_1	105.09 (7)	N1_2—C1_2—C2_2	105.10 (7)
N1_1—C1_1—H1_1	127.5	N1_2—C1_2—H1_2	127.5
C2_1—C1_1—H1_1	127.5	C2_2—C1_2—H1_2	127.5
F1_1—C2_1—C1_1	125.91 (7)	F1_2—C2_2—C1_2	126.25 (7)
F1_1—C2_1—C3_1	126.73 (7)	F1_2—C2_2—C3_2	126.46 (7)
C1_1—C2_1—C3_1	107.36 (7)	C1_2—C2_2—C3_2	107.29 (6)
N2_1—C3_1—C2_1	109.29 (7)	N2_2—C3_2—C2_2	109.46 (7)
N2_1—C3_1—H3_1	125.4	N2_2—C3_2—H3_2	125.3
C2_1—C3_1—H3_1	125.4	C2_2—C3_2—H3_2	125.3
			
C1_1—N1_1—N2_1—C3_1	0.49 (9)	C1_2—N1_2—N2_2—C3_2	0.85 (9)
N2_1—N1_1—C1_1—C2_1	−0.31 (9)	N2_2—N1_2—C1_2—C2_2	−0.88 (9)
N1_1—C1_1—C2_1—F1_1	179.71 (7)	N1_2—C1_2—C2_2—F1_2	−178.67 (7)
N1_1—C1_1—C2_1—C3_1	0.01 (9)	N1_2—C1_2—C2_2—C3_2	0.55 (9)
N1_1—N2_1—C3_1—C2_1	−0.46 (9)	N1_2—N2_2—C3_2—C2_2	−0.46 (9)
F1_1—C2_1—C3_1—N2_1	−179.41 (7)	F1_2—C2_2—C3_2—N2_2	179.16 (7)
C1_1—C2_1—C3_1—N2_1	0.29 (9)	C1_2—C2_2—C3_2—N2_2	−0.06 (9)
